# The feasibility of wildlife immersion experiences for Veterans with PTSD

**DOI:** 10.3389/fvets.2024.1290668

**Published:** 2024-05-30

**Authors:** Donna J. Perry, Sybil L. Crawford, Jill M. Mackin, Jesse J. Averka, David A. Smelson

**Affiliations:** ^1^Tan Chingfen Graduate School of Nursing, UMass Chan Medical School, Worcester, MA, United States; ^2^Soldier On, Pittsfield, MA, United States; ^3^Department of Psychiatry, UMass Chan Medical School, Worcester, MA, United States

**Keywords:** feasibility, human-animal interaction, human-wildlife interaction, animal-assisted intervention, Veterans, PTSD, transcendent pluralism, wildlife immersion

## Abstract

**Introduction:**

Animal-assisted interventions (AAI) offer potential physical and psychological health benefits that may assist Veterans with post-traumatic stress disorder. However, more feasibility studies are needed regarding intervention details, adverse events, reasons for study withdrawal, and animal welfare.

**Methods:**

This mixed methods feasibility trial involved a modified crossover study in which Veterans with PTSD/PTSD symptoms were provided a series of 8 nature and wildlife immersion experiences to evaluate feasibility and preliminary efficacy. The sample included 19 Veterans with PTSD/PTSD symptoms who were followed for a mean of 15.1 weeks. The intervention was comprised of a baseline forest walk, assisting with wildlife rehabilitation, observation in a wildlife sanctuary, and bird watching. Post study bird feeders were provided for sustainability. The theory of transcendent pluralism, which is grounded in mutual human and ecological dignity, guided the study. We viewed feasibility from the perspective of pattern integration with the natural world.

**Results:**

This AAI nature/wildlife immersion intervention was feasible, acceptable, and safe to administer to Veterans with PTSD/PTSD symptoms with appropriate support. Logistical and relational facilitators were identified that supported the wildlife immersion activities. Participants reported greatly enjoying the activities. Attention to animal welfare and care was an important ethical foundation that also contributed to feasibility.

**Discussion:**

AAI immersion experiences with wildlife are feasible and can safely be administered to Veterans with PTSD/PTSD symptoms. Logistical and relational facilitators are important to support nature and wildlife immersion activities.

## Introduction

Human-animal interaction (HAI) encompasses many areas of study (1). In the context of health care, HAI focuses on the ways in which encounters between humans and other species enhance human physical and/or psychological well-being (2). Within the field of HAI, an animal-assisted intervention (AAI) is defined as “a goal oriented and structured intervention that intentionally includes or incorporates animals in health, education and human services (e.g., social work) for the purpose of therapeutic gains in humans” (3), (p. 5). Types of AAI include therapy, education, activities, and coaching (3). A meta-analysis of 49 studies with animal-assisted therapy found improved outcomes with moderate effect sizes in medical conditions, behavioral problems, emotional well-being, and autism-spectrum symptoms (4).

One of the areas in which AAI has shown promise is for persons with post-traumatic stress disorder (PTSD). Approximately 6% of the U.S. adult population experiences PTSD at some point in their lives. Lifetime prevalence rates are 7% in Veterans and as high as 29% among those that served in Iraq and Afghanistan (5). Veterans with PTSD face a higher risk of isolation, homelessness, substance abuse, suicide, and stigma about getting help (6, 7). Animal-assisted interventions may circumvent stigma due to “the nonjudgmental nature of human-animal interactions” (6), (p. 55). A systematic review of AAI in 10 studies showed improvement in PTSD symptoms as well as levels of depression and anxiety (8).

However, some of the literature with AAI suggests the need for further methodological development. Broad qualitative and quantitative approaches are needed to capture the complexity of HAI (9). HAI research must provide more detailed information regarding interventions (10, 11), best practices (10), animal welfare considerations (10, 12, 13), adverse events, and reasons for study withdrawal (14).

Most research with HAI has focused on domesticated animals (companion and agricultural) (1), although there is some literature showing potential benefits from human interaction with wildlife (15–17). A growing body of literature also supports favorable health benefits from nature contact itself (18, 19). The term “green care” has been proposed as a concept that encompasses the health benefits from natural resources, including animals (20). As with domesticated animals, however, human-wildlife interactions can pose risks to health such as zoonotic diseases (12, 21). It is critical for research to identify the conditions under which such interactions might be beneficial and safe.

We conducted a pilot feasibility study to examine the influence of animal-assisted activities with wildlife for Veterans with PTSD/PTSD symptoms. This study builds on recent research by the principal investigator (PI) on human-wildlife interactions (22, 23). The overall study purpose was to assess feasibility and preliminary efficacy of the intervention. Feasibility studies are helpful when there are few studies on a particular intervention to determine whether the intervention is practical in a real-world setting and appropriate for further testing. Feasibility studies may also include an experimental design to address the question, “Can it work?” (24), (p. 4). The purpose of this paper is to address findings from the first study aim to determine the feasibility, safety and acceptability of an AAI intervention comprised of wildlife immersion activities for a sample of Veterans with PTSD/PTSD symptoms.

## Materials and methods

### Study overview/human subjects protection

The intervention was a nature/wildlife immersion for Veterans with PTSD/PTSD symptoms which was designated as an animal-assisted activity. Animal-assisted activities are a type of AAI which involve informal goal-oriented interactions for purposes of education, motivation, and recreation (3). We utilized a modified crossover design in which participants engaged in a series of wildlife immersion activities within groups of 2–8 individuals. Institutional Review Board (IRB) approval was obtained from the PI’s university (# H00016795). Participants were provided with a $30 gift card after each of the first seven activities, a $100 gift card after the final activity, and a $30 gift card after the follow up interview. Sustainability was built into the study through providing each participant with a home birdfeeder and supplies. At the conclusion of the study, participants were also provided with the study wrist monitors to keep. Data were collected from July 2019 to December 2022. The study was on hold from March 2020 until August 2021 due to the Covid pandemic, which limited access to study sites.

### Theoretical perspective

The study was guided by the PI’s theory of transcendent pluralism which is grounded in mutually evolving human and ecological dignity (25). This framework has been influenced by the philosophy of Bernard Lonergan, Native American teachings, and a unitary-transformative nursing perspective (26–28). It had originally been used to study relations between diverse groups of people but has recently been expanded to explore relations between humans and other species. In transcendent pluralism, pattern is the expression of an individual or group’s way of being in the world and includes consciousness and behavior (25). Patterns are dynamic and can evolve over time.

From this lens, the central questions for a feasibility study explore how the patterns of the study participants can be integrated with natural and organizational patterns of the study activities. Will the participants choose to engage in these new patterns (recruitment)? Which aspects of the intervention do they find agreeable (acceptability) and how consistently will they participate in the intervention (retention)? In what ways do they choose to integrate new patterns in their lives after the study is over (sustainability)? And how can these patterns of the intervention be implemented in such a way that supports the dignity of humans and wildlife, including physical safety and emotional well-being?

### Recruitment

Veterans with PTSD/PTSD symptoms were recruited through Soldier On, a non-Veterans Administration community partner, which has a residential facility serving Veterans in Western Massachusetts. Soldier On is a non-profit organization dedicated to ending Veteran homelessness by providing temporary and permanent housing. Each Veteran that enters Soldier On is provided with supportive services including a case manager with whom participants typically discussed study enrollment. Inclusion criteria were: PTSD/PTSD symptoms (per self-report), age 18–70, comfortable interacting with animals, ability to walk or use wheelchair up to one mile at a leisurely pace, cognitive ability to complete assessments, vision or hearing impairments (if present) corrected through glasses and/or hearing aid, service animals allowed within behavioral parameters, free of substance abuse for at least 30 days, stated willingness to refrain from drug/alcohol use during activities, no severe outdoor allergy, and not presently enrolled in Veterans’ Treatment Court program. During the late Covid phase, requirements were added for Covid vaccination. Participants were recruited through flyers, word of mouth, and referral by case manager. A Veteran at Soldier On undertook a research assistant role to facilitate recruitment. The PI also went to the facility to attend community meetings and explain the study. The PI met with each participant to obtain consent and complete baseline data collection.

### Setting

The intervention was provided at four community partner organizations with a strong history of introducing members of the public to nature and wildlife, safe/accessible sites, and experts on staff to provide education. The PI had an established relationship with two of the organizations from prior research. The travel time to each location ranged from 1 to 4 h. Transportation was conducted via vans owned by Soldier On or by chartered buses. Participants were provided with lunch and snacks for the ride home.

### Intervention

The intervention consisted of a series of 8 nature/wildlife immersion experiences provided in four different settings. By “immersion experience” we mean that study participants were invited into natural settings and wildlife-rich environments. We define a wildlife immersion activity as *an embodied spatial–temporal experience in which human participants enter a space (setting) in which they consciously engage with wildlife in a manner that affirms the dignity of both the human and beyond-human animal with respect for natural rhythms.*

Each setting had some consistency in general patterns but actual conditions such as weather and animal availability varied on a day-to-day basis. The first activity was an introductory forest walk to control for confounding effects of nature alone followed by 3 wildlife activities. The immersion experiences included: baseline woodland walk (Harvard Forest); assisting with wildlife rehabilitation care (New England Wildlife Center); observation of wildlife sanctuary care/enrichment (Maine Wildlife Park); and bird watching/raptor program (Mass Audubon Broad Meadow Brook Conservation Center and Wildlife Sanctuary/Massachusetts Bird of Prey Rehab Facility).

Each immersion experience included a didactic education component as well as a nature/wildlife activity and was conducted at two time points to reduce novelty effect. The immersion experiences were held approximately 1 week apart and were of 3–4 h in duration. Due to the intervention being conducted in New England, no activities were held during the winter. Standard operating procedures were developed for the immersion intervention at each study site (Table 1). Participants were invited but not required to participate in activities at the sites.

**Table 1:** Sample standard operating procedure: Maine wildlife park (MWP).

**Arrival logistics/preliminary data collection**
PI arrives ½ h ahead of participants and sets up study materials on picnic table.
Participants driven to park from Soldier On; scheduled to arrive 15 min prior to activity for bathroom use etc.
Participants given name tags and asked to write first name.
Participants should have their wrist monitors with them.
Participants asked to record their heart rate after a 5-min rest.
On second visit: Participants given salivary cortisol packet and asked to obtain sample (discontinued during COVID period).
Participants reminded to apply tick spray and to check for ticks after activity.
Introduction given for morning speaker.
**Intervention**
*MWP Visit 1*
(Education and guided walk provided by Gamekeeper and Educational Specialist)
Educational overview of MWP and animal husbandry in wildlife care (approximately ½ h)
Wildlife immersion activity: Guided walk viewing different species in sanctuary (approximately 1–1.5 h)
*MWP Visit 2*
(Education and guided walk provided by Gamekeeper and Educational specialist)
Educational overview of animal enrichment rationale and different strategies for animal enrichment (approximately ½ h)
Wildlife immersion activity: guided walk viewing enrichment activities with animals (approximately 1–1.5 h)
Follow-up data collection
Participants walk back to picnic table.
Participants given data sheets and heart rate forms on clipboard with pen.
Asked to record heart rate after 5-min rest.
On second visit: participants given salivary cortisol packet and asked to obtain sample (discontinued during COVID period).
Participants asked to complete survey forms.
Participants asked to complete journal forms (participants asked to choose animal that was meaningful to them and to write down insights gained; option of sitting at table to write their insights or walking back to sit near animal).
Lunch break/free time
Closure/departure
Departure in van/bus; Snack stop on way home.

### Animal welfare

Animal welfare must be at the forefront of AAI practice and research (10, 29). When AAI is being conducted for human benefit, “there is an ethical obligation for these animals to achieve ‘very good’ welfare status” (29), (p. 56). Animal welfare includes being free of distress and able to engage in the natural behaviors of each species (29). This is congruent with the theory of transcendent pluralism in which ‘non’ human animals are viewed as having intrinsic value and are not merely human instruments (23). The intervention was grounded in mutual human and beyond-human dignity. Wildlife in this study were defined as, “non-domesticated amphibians, reptiles, birds, and mammals” (30). This included both free-living creatures as well as those living in temporary or permanent captivity due to animal care needs. The missions of the partnering wildlife facilities in this study were focused on animal welfare through direct animal care and/or public education. All the activities that participants observed or engaged in were standard activities for the wildlife under care. A wildlife veterinarian served as a consultant to the study.

Spatial territory is a core need for wildlife and must be respected during AAI to avoid animal stress and maintain human safety. Engagement in shared wildlife space requires attentiveness to one’s own spatial relationship with the animal (22, 31). The study intervention was guided by experts at each location so that appropriate boundaries, protocols, and assessment for animal stress were maintained. Activities were framed within the natural patterns of each species within its environment with education provided to help participants understand these rhythms.

### Safety

A robust data safety and monitoring plan was instituted prior to study enrollment due to the risks of bringing individuals with both psychological and medical conditions into nature/wildlife immersion settings. Risk examples and associated strategies are outlined in Table 2. The PI, a registered nurse and wildlife rehabilitator, attended all study activities, carried a first aid kit, and was certified in wilderness first aid.

**Table 2:** Risk reduction measures (examples).

Risk examples	Protective measures
Animal bite or scratch	Education prior to activities. Wearing gloves; long sleeves recommended. Availability of first aid kit for all activities. Follow up medical care available at VA.
Exposure to zoonotic disease	Follow facility protocols. Use of handwashing/gloves in wildlife hospital. No handling of raccoons (rabies vector). Exclusion of individuals with severe immunocompromised status.
Exacerbation of psychological symptoms	Ongoing assessment by PI. Action plan for acute referral if needed.
Substance abuse exacerbation during activity	Inclusion criteria of 30 days or more free of drugs or alcohol abuse and willingness not to use substances before or during activities. Narcan nasal spray included in first aid kit.
Tick bite during outdoor walk	EPA approved tick sprays provided to participants with reminders for application. CDC education sheet on tick bites provided with instructions to monitor for tick bites following activity and f/u with MD if needed.

The emergence of the Covid-19 pandemic necessitated additional safety modifications. During 2020 and the first half of 2021, the study had to be put on hold because Covid-related restrictions prevented access to some activity sites. When the study resumed, we made modifications to reduce the likelihood of Covid transmission. This included requiring individuals to be fully vaccinated against Covid or, if partially vaccinated, to complete a home antigen test the morning of each activity and to text the results to the PI. While this involved some early morning logistics, there was 100% compliance, and we had no cases of Covid transmission. Although the vaccine requirement did impact recruitment (at least 2 who did not enroll due to vaccine), it was critically important due to participants with significant medical comorbidities engaged in group activities and travelling in a single vehicle. We also eliminated the salivary cortisol samples as these were being collected in a group setting.

### Data collection/analysis

Feasibility evaluation included recruitment, enrollment, attendance/retention, adverse events, missing data, responses to a post activity survey, a focus group immediately after the final event (Table 3), individual interviews approximately 1 month later (Table 4), and observation by the PI. The PI maintained an observation journal and safety assessment was ongoing. In order to determine preliminary efficacy, psychological well-being was assessed at baseline (during enrollment; approximately 1–2 weeks prior to first activity) and after each activity through: Warwick-Edinburgh Mental-Well Being Scale (32, 33), Spielberger State/Trait Anxiety Inventory (short-form) (34), and Center for Epidemiologic Studies Depression (CES-D-10) scale (35). PTSD symptoms were evaluated through a PCL-5 at baseline and study conclusion (36, 37). Connection to nature and wildlife were measured at baseline and after each activity through the short-form Nature-Relatedness scale (NR-6) (38) and Transcendent Feelings of Animal Valuation Scale (23). Physiological parameters included self-monitored heart rate at baseline and pre and post each activity using commercial wrist monitors. Self-monitoring was selected so that all participants’ heart rates could be obtained simultaneously. Salivary cortisol was measured at baseline and before/after the second activity at each site with the first 12 participants. Qualitative assessment also included an animal observation journal exercise during the Maine Wildlife Park activities.

**Table 3:** Focus group flexible interview guide (conducted by PI with each group at study conclusion).

1. How was this experience for you as a whole?
Probe: What are some of the most memorable of the activities for you?
What sorts of questions did you have as you were going through this experience?
Probe: Did the wildlife activities make you wonder about any new things?
What knowledge did you gain from this experience?
Has this experience led you to view anything differently?
Has this experience led you to take any new actions? If so, what?
How did this program influence your relationships with other Veterans in your group?
How has this experience been meaningful in your life?
Is there anything else you would like to add?

**Table 4:** Follow-up flexible interview guide (conducted by PI 4–6 weeks after study conclusion).

Could you please tell me how you found your experience with the wildlife program as a whole?
Could you think back to one of the times you observed or interacted with an animal that was particularly memorable. What made that experience meaningful for you?
How was your experience of doing the activities with the group?
Could you please tell me if you are using the home bird feeder? (yes/no)
How often do you look at the bird feeder on an average day?
1× day___; 2–3 times per day___; 4 or more times per day___
How long do you spend watching the feeder each day? (in minutes)
How do you feel when you watch the birds?
Do you feel that connecting with wildlife is beneficial to you in any way and, if so, how?
Have you done any other wildlife activities since the program ended?
Do you plan to engage in any wildlife activities in the future? If yes, what activities?
Is there anything else that you want to add?

Quantitative data analyses included tabulations of number of potential participants screened, number screened who were eligible, number of eligible who enrolled, and reasons for non-enrollment. We summarized characteristics of participants using frequencies for categorical characteristics and mean and standard deviation for continuous characteristics. To summarize attendance at wildlife immersion activities, we calculated within-participant percentage and mean and median number of activities attended, as well as the percentage of all participants attending for each activity. We also tabulated reasons for non-attendance. We tested whether attendance was different at the first and second sessions of each activity using McNemar’s test (39). Associations of participant characteristics with study completion and with number of activities attended were assessed using Fisher’s exact test and Wilcoxon 2-sample tests, respectively. To assess acceptability, participants evaluated each activity directly after its conclusion, using six 5-point Likert scales (1 = strongly disagree, …, 5 = strongly agree) regarding enjoyable, adequate instructions, stressful (reversed for analyses), felt good, would like to do again, and recommend to others. Ratings on each of the 6 aspects were averaged to provide a summary score from each participant for each activity attended (Cronbach’s alpha = 0.85). Based on the observed distribution – minimum of 3.7 – these average scores were dichotomized as strongly agree (4.5 or higher) versus agree (<4.5). Activities were compared regarding percent of participants with a strongly agree rating using binomial logistic regression with a random effect for participant (40), adjusting for first versus second session of the activity. In addition, directly after each activity’s conclusion, participants also reported satisfaction with the amount of time spent (just right, too long, too short). Finally, for attended activities, we tabulated whether the participant completed all data collection measures. Quantitative findings related to preliminary efficacy will be reported elsewhere.

Focus groups were audio recorded using two devices and analyzed by a secure transcription service. The PI listened to the tapes and edited as needed. The follow up interviews were transcribed from handwritten notes by the PI. Survey responses to open-ended questions were entered into Word documents and organized into tables. All text documents were read multiple times with topical codes developed. Responses related to feasibility were analyzed using relevant pre-existing categories, such as safety (41). Responses related to preliminary efficacy were analyzed separately using open-ended coding (41) and will be reported separately. The qualitative and quantitative feasibility findings are integrated below. Quotations from the participants are included to illustrate the findings with extraneous words such as “like” removed for clarity. Lincoln and Guba’s standards for qualitative trustworthiness were followed including credibility (triangulation of data, prolonged engagement in field); transferability (rich description; purposive maximum variation sample); dependability (audit trail), and confirmability (audit trail, reflexivity) (42).

## Results

### Recruitment and retention (will participants choose to engage in new immersion patterns?)

Twenty-nine individuals were screened for the study. Of these, 28 were deemed eligible and one was not eligible due to being unvaccinated for Covid. Another individual indicated interest but was not vaccinated and did not sign up for screening. Out of those eligible, 9 subsequently did not enroll due to issues related to health (3), left Soldier On (1), logistical issues (1), or study placed on hold due to Covid (4).

Nineteen participants were enrolled in the study within 4 cohorts and together completed a total of 107 wildlife immersion experiences across 32 project activity sessions, 2 per activity location and 8 per cohort. One cohort was planned as an all-women’s group due to some history of military sexual trauma in the population. Another group was coincidentally all women, and two groups were mixed. The mean study duration for participants who completed the study was 15.1 weeks from enrollment to follow up interview. Demographics are summarized in Table 5. Military branches included Army, Air Force, Navy, and Marines. We omitted the branches in the table tabulations in order to avoid any participant identification due to low cell count in some categories. In addition to PTSD, the majority of the participants had co-existing medical and psychiatric conditions. Three participants used walkers or canes and one required portable oxygen.

**Table 5:** Participant baseline characteristics, *N* = 19.

Characteristic	*N* (%) or Mean (Std. Dev)/Minimum – Maximum
Age in years	48.4 (12.9)/30–69
*Gender*	
Female	13 (68.4)
Male	6 (31.6)
*Race*	
Black	3 (15.8)
White	16 (84.2)
*Residence*	
Permanent/committed plan for permanent	12 (63.2)
Short-term	7 (36.8)
*Medical history*	
Cardiovascular disease	4 (21.1)
Respiratory disorder	4 (21.1)
Orthopedic/movement condition	7 (36.8)
Diabetes mellitus	3 (15.8)
Psychiatric disorder	14 (73.7)
*PCL-5^a^*	
Total score	41.0 (18.1)/7–65
Score ≥ 31	14 (73.7)
*STAI*	
Total score	50.5 (11.4)/20–70
Score > 36	18 (94.7)
*CES-D-10*	
Total score	15.3 (5.2)/5–23
Score ≥ 10	16 (84.2)

^a^Range 0–80 with 31–33 as cut-off score for provisional PTSD diagnosis.

**Figure 1:**
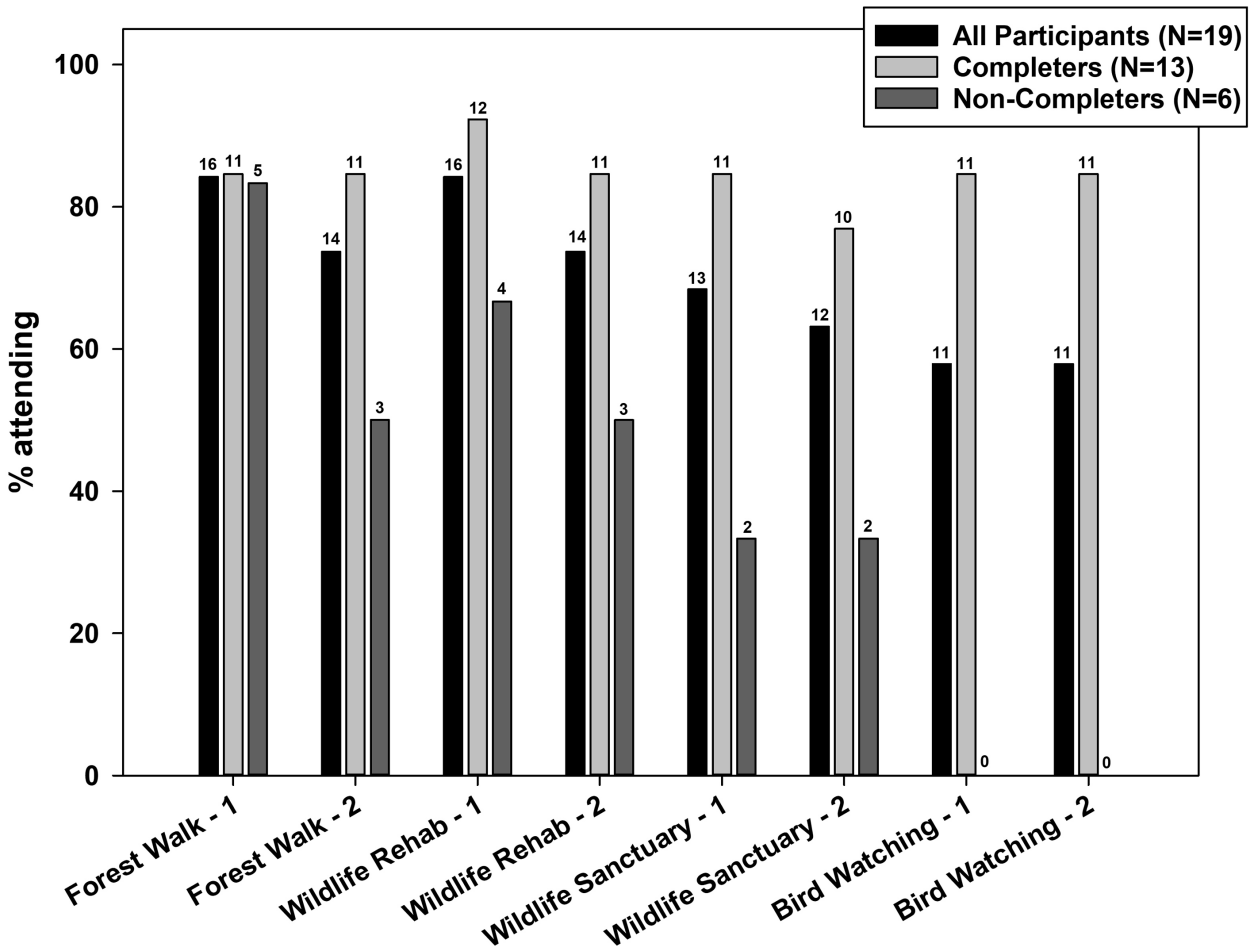
Attendance at wildlife immersion experiences.

Participants attended an average of 5.6 out of 8 study activities (median 7). Twelve participants (63.2%) completed at least 6 activities. Attendance ranged from 84.2% at the first activity to 57.9% at the eighth and last activity (Figure 1). Although second-session attendance was lower than at the first session for the first three project activities, none of these differences was statistically significant (*p* > 0.3173). During 2019, when there were two cohorts, participants who missed an activity in Cohort 1 were offered the opportunity to attend the activity with Cohort 2. Participants did choose to make up a session on 4 occasions. Five participants (26.3%) withdrew from the study, accounting for 14 missed study sessions (Table 6). Four of these withdrew due to leaving Soldier On and moving to other geographical locations. Another individual did not formally withdraw but attended only one activity (data from this participant was retained because omission did not change overall results). No participants indicated that they withdrew for study-related reasons. In addition to study withdrawal (accounting for 9.2% of 152 (=19 participants × 8 activities) scheduled study activities), most common reasons for missing an activity were illness (3.9%), a health care appointment (4.6%), and unknown/no-show (6.6%). Median number of activities attended (Table 7) was higher in female than male participants (7 versus 3, *p* = 0.06) and in Black than White participants (8 versus 6, *p* = 0.07). Study completion (Table 7) did not differ significantly by any participant characteristics. Three activities were rescheduled due to rain, and most participants were able to attend the new date.

**Table 6:** Reasons for non-attendance; across all 8 sessions per cohort and all 19 participants, 152 scheduled participant-sessions in total.

Reason	*N* (%) reporting reason
Withdrew from study	14 (9.2)
Unknown; no-show	10 (6.6)
Health care appointment	7 (4.6)
Illness	6 (3.9)
Work-related	5 (3.3)
Not interested	0 (0.0)
Other	7 (4.6)

**Table 7:** Study completion and number of activities attended; all participants and by participant characteristics.

	Study completion			Total number activities attended, all participants (*N* = 19)	
	Completers: *N* (%) or Median (IQR)^a^	Non-Completers^d^: *N* (%) or Median (IQR)^a^	Test statistic/*p*-value^b^	Median (IQR) or Spearman correlation^a^	Test statistic/*p*-value^c^
*Characteristic*					
All participants	13 (68.4)	6 (31.6)	–	7 (3, 7)	–
*Gender*			0.2108/0.3201		37.00/0.0587
Female	10 (76.9)	3 (23.1)		7 (6, 7)	
Male	3 (50.0)	3 (50.0)		3 (2, 6)	
*Race*			0.2951/0.5170		47.50/0.0677
Black	3 (100.0)	0 (0.0)		8 (7, 8)	
White	10 (62.5)	6 (37.5)		6 (3, 7)	
*Residence*			0.0851/0.1287		56.50/0.2759
Permanent	10 (83.3)	16.7 (2)		7 (5.5, 7.5)	
Short-term	3 (42.9)	57.1 (4)		4 (3, 7)	
*Age*	56 (42, 59)	34.5 (30, 48)	40.00/0.1041	0.26	0.2631/0.2765
*Baseline PCL-5 ≥ 31:*			0.3689/1.0000		57.00/0.5460
No	4 (80.0)	1 (20.0)		7 (6, 8)	
Yes	9 (64.3)	5 (35.7)		6.5 (3, 7)	
*Baseline STAI >36*			0.6842/1.0000		17.50/0.2077
No	1 (100.0)	0 (0.0)		8 (8, 8)	
Yes	12 (66.7)	6 (33.3)		6.5 (3, 7)	
*Baseline CES-D-10 ≥10*			0.2951/0.5170		42.50/0.1869
No	3 (100.0)	0 (0.0)		7 (7, 8)	
Yes	10 (62.5)	6 (37.5)		6 (3, 7)	
*Study completion*			–		25.50/0.0069
Yes	13 (100.0)	0 (0.0)		7 (7, 8)	
No	0 (0.0)	6 (100.0)		3 (2, 4)	

^a^IQR = interquartile range (25th percentile, 75th percentile). ^b^Fisher’s exact test (test statistic presented is table probability) or Wilcoxon 2-sample test for age. ^c^Wilcoxon 2-sample test for 2 subgroups, Kruskal–Wallis for 3+ subgroups, Spearman correlation for continuous characteristics. ^d^Non-completers includes 5 participants that withdrew from the study as well as 1 who attended only one event but declined to withdraw.

Regarding completion of specific protocol components, of the 107 activities attended, pre-activity pulse was measured in 98.1% and post-activity pulse in 96.3%. Among Cohort 1 and 2 attendees, participants completed 81 of 84 (96.4%) cortisol specimen collections (goal was 1 at baseline, 2 at the second session of each wildlife immersion activity per participant); of the 81 specimens collected, 74 (91.4%) had adequate volume. Across the eight activities, the median percent of missing survey items was 5.2% (interquartile range 3.5, 6.9%).

Some participants indicated that recruitment for a long-term program among this population was difficult, especially since many individuals were at a point of trying to stabilize their lives.

… You’re looking at a place where the turnaround of people is incredible. You have people coming in and out… People have appointments… They’re out looking for jobs… So that's what makes it hard for- a program for anybody to commit to.

### Acceptability (will immersion experiences be positively perceived by participants?)

With the exception of one response (=3.7), all session evaluations were rated as agree or higher. The percentage of participants reporting strongly agree ranged from 70% for bird watching to 84% for wildlife sanctuary (adjusted *p* = 0.72). Percent reporting strongly agree also did not differ for first versus second session of an activity (77% versus 78%, adjusted *p* = 0.995). Across all activities attended, 93.3% (*n* = 98) participants reported that the activity time was just right. Only 1% (*n* = 1) reported too long, and 5.7% (*n* = 6) reported too short (rating missing for 2).

In qualitative assessments, participants’ reception of the study was overwhelmingly positive. They described the activities as something they “looked forward to” and “loved every part of it.” Several commented on how “it was amazing to see the animals up close…” As one participant noted, “It was very exciting. -I never really had a chance, being in the city, to focus on birds and their sounds.” Several participants expressed sadness that the study was ending and asked if they could continue or re-enroll. For example, a participant shared:

The whole experience… has given me something *good* to look forward to each week. And knowing that we’re gonna go do another activity…. Has been *up*lifting. It’s given me something exciting to think about. And it's been a lot of fun. So, I’m kind of sad that it's coming to an end.

Another stated, “Most of us wish it could have gone on longer and longer and longer.” A number of participants noted that they enjoyed having different activities to participate in. “You learn something new when it’s not repetitive”.

Participants seemed very amenable to completing the surveys after each activity and collecting samples for salivary cortisol. Having participants monitor and record their own pulse pre and post activity worked very well, and several seemed to enjoy noting changes in their heart rate.

### Safety and wellbeing (can interventions support dignity and wellbeing of both participants and wildlife?)

#### Safety occurrences

Safety incidents were reviewed and categorized as mild, moderate, or severe and as not related, possibly related, or related to the study. A few safety incidents did occur that were all in the mild or moderate category with no long-term sequelae experienced. Four of these were deemed related/possibly related to the study intervention and all resolved with no or minor intervention (Table 8). These included two participants who found ticks (American dog ticks, not embedded) on their skin after a forest walk (likely related to a parking/drop off area near high grass), a participant with Type II diabetes who reported mild hypoglycemic symptoms walking in the forest, a participant who experienced an acorn falling on her shoulder, and minor scratches on a participant’s arm after feeding baby squirrels.

**Table 8:** Intervention-related/possibly-related safety events.

Incident	Severity level	Related to study?	Action taken
Two participants found ticks on themselves after forest walk (American dog ticks; not embedded).	Mild	Related	Reinforcement of tick prevention education. Earlier application of tick spray. Provision of multiple options for tick spray (EPA approved). Education regarding checking for ticks after activities. Changed parking location away from high grass.
Participant with Type II diabetes reported feeling slightly hypoglycemic during forest walk.	Moderate	Possibly related	Individual improved after eating candy. Added hard candy to activity first aid kit.
Minor scratches to participant’s arm after feeding baby squirrels.	Moderate	Related	Individual washed arms and applied antiseptic; no sequelae.
Participant hit by falling acorn.	Mild	Related	Resolved without intervention.

We were able to safely include individuals with moderate physical limitations through strategies such as having a registered nurse (PI) present at all study activities, obtaining baseline medical histories to be aware of needs, and providing all-terrain wheelchairs (Grit Freedom Chair). There were three individuals who used walking implements such as a walker or cane. Two participants used wheelchairs on the first activity but only periodically after that, such as on a steep incline. One individual chose to use the wheelchair as a walker, and occasionally using it to sit and rest. Another participant preferred to use their own walker, but the PI followed with the wheelchair as a backup. Although participants did not use the wheelchairs all the time, having them available seemed to give individuals the confidence to enroll in the study. Honoring their decision to choose the method of assistance was important for respecting their ability to make their own choices. Participants navigated the terrain successfully. The pace was generally slow due to frequent education stops but the PI also carefully assessed activity tolerance and suggested a rest periodically. Other physical conditions that we were able to accommodate included portable oxygen use which required that participant to plan how many tanks would be needed for the activities.

Safety assessment was important at the wildlife rehab center where participants had the option to engage in direct animal care. This included feeding baby squirrels via syringe, feeding birds of prey, releasing animals whose treatment had finished, assisting with reptile examinations, and administering medication under the guidance of veterinary staff. Other than one mild arm scratch, there were no safety incidents. No animal stress was noted. On one occasion an interactive experience with an educational red-tailed hawk was postponed because staff assessed that he was not in a “good mood.”

In accordance with study protocol, we notified the case manager if any participants had a high baseline score for anxiety or depression at study enrollment (as defined in literature) or a > 25% increase over baseline anxiety or depression scores during post intervention measurements. Communication of score elevations allowed case managers to assess whether results were clinically relevant, and address as needed. Case managers expressed appreciation for this communication. For the CES-D-10, the range is 0–30 and a score of 10 or higher indicates significant depression symptoms (43). The range of the STAI-Y-6 item is 20–80 with 34–36 considered normal (35, 44). Baseline anxiety scores were elevated for 18 of 19 participants and baseline depression scores were elevated for 16 of 19 participants. Anxiety elevations required notification for 3 participants (on 3 occasions for one participant). Depression score notifications were required for 5 participants (on 2 occasions for 1 participant). Participants’ qualitative comments and general demeanor did not suggest that the elevated scores related to the wildlife activities. During one post study focus group, participants spontaneously noted that stressful events in their day-to-day lives were impacting their survey responses. It seemed likely that the high anxiety and depression scores reflected baseline diagnoses in concert with ongoing extraneous events rather than study activities. Of note is that no illicit substance abuse issues were manifested during any study activities.

#### Detractors/stress/anxiety

Participants were queried as to whether anything in the activity or surrounding area made them feel stressed or anxious. Some of the comments noted that challenges such as rocks or rough terrain when walking and insects such as ticks or mosquitos created stress. An individual with underlying respiratory disease noted some stress due to being short of breath while walking but that this was “normal for me.” In the post activity Likert scale, across all 8 wildlife immersion activities, there were only 2 reports that an activity was stressful (*n* = 2).

We did not expose participants to euthanasia situations, but they were told that the injuries sustained by wild animals were sometimes not survivable. We informed participants in the consent form that they might be exposed to deceased and dying animals. In a few instances, participants witnessed deceased animals. This included taxidermy specimens during an educational presentation and a deceased bird on an outdoor walkway, likely due to an accidental window strike. No participants indicated stress from these instances. Two occasions which participants did note as stressful were seeing deceased animals, such as guinea pigs, when they were assisting in the food preparation area (diet for birds of prey) and finding a deceased squirrel in its enclosure. In both instances, the stress seemed to be transient. Two individuals participated in optional opportunities to feed deceased prey to raptors, with positive responses.

Although one participant felt that the long ride (4 h) to an out of state activity was too long, most participants felt that the drive was “worth it,” and another participant reported that this activity was too short. We adjusted this activity by using commercial buses rather than vans and participants evaluated that trip as comfortable and enjoyable.

We tried to anticipate environmental stressors such as communicating with a local gun club to avoid holding research activities when large shooting events were scheduled. However, the environment could intrude in unexpected ways that could not be controlled. For example, while in a bucolic Maine park during autumn, participants were startled by acorns suddenly falling with a rat-a-tat sound upon the pavilion roof. A nearby screaming child was also noted as disruptive on a participant evaluation.

Experiences held different meanings for different participants. When we came upon a research tower during the forest walk, one participant seemed to regard it as an adventure and was disappointed that climbing the tower was not allowed. Another participant indicated that the tower was stressful as it reminded them of military training.

### Relational immersion facilitators

In addition to the logistical aspects of the study that enhanced feasibility, as described, above the findings suggest that positive nature and wildlife immersion experiences were facilitated by relational dimensions. This included the group-based intervention, community partners, animal welfare mission, research team support, and responsiveness to needs.

#### Group intervention

Qualitative findings as well as observation suggested that the group-based intervention enhanced recruitment, retention, and acceptability. Participants were living in group housing and individuals encouraged each other to join the study and reminded each other to attend activities. Participants found that sharing the experience with others was valuable. “When you had more than one person, you are able to talk to the other people… to see how they felt about the other animals”.

Group members supported one another during activities. For example, a participant that had successfully fed several baby squirrels encouraged a more hesitant individual to give it a try and stood next to her as she did the feeding. At one point on the trail, participants spontaneously helped one another, such as by carrying another person’s walker while that person used the wheelchair. The groups seemed to develop a cohesive spirit, perhaps reflecting their background as Veterans and unified military culture. Some participants indicated that the group activities promoted friendships and deepened relationships during the study and may have contributed to ongoing benefits after study conclusion. “I liked it in a group because everybody got to ask different questions. Afterwards somebody to talk about it with. ‘Remember that time we saw the raccoons?’”

There were some minor challenges with the cohort model including occasional tension between group members that may have been related to differences in age and rehab status as well as time delays by individuals requesting a smoking break. There was also a logistical challenge when one individual experienced health issues during a forest walk, which slightly shortened the walk for others. Some participants had a history of social anxiety, and one individual expressed a need for solitude. Time away from the group was accommodated by giving the participants free time at lunch to explore and an option to write their animal reflection journal by themselves in front of the animal or with the group at the table after lunch. Overall, these challenges were minor, and the group model was largely perceived positively.

#### Community study partners

Recruiting participants from an organizational Veterans’ program provided a solid foundation for participant support. We worked closely with Soldier On leadership, case managers, and psychiatric staff to ensure that participants were well supported during the program. Additionally, staff at each site were instrumental in providing background information, concrete activity instructions, and positive reinforcement. All the staff utilized an interactive format to actively engage participants in learning. This included ample opportunity to ask questions. Participants appreciated gaining knowledge and support from staff was instrumental in trying out new activities. For example, during the forest walk a staff member coached participants in how to measure tree trunk diameter, affirming afterwards, “That’s perfect.” At the wildlife hospital, the veterinarian coached participants in feeding a baby squirrel by demonstrating how to hold the animal and providing pointers on using the formula syringe. One participant described how the veterinarian’s encouragement helped her overcome fear of holding a boa constrictor.

The most memorable was the snake. Being able to actually hold it… That was big for me. I have a new appreciation. Because I was not going to. That vet said, “Come on”. I was surprised. It was relaxing.

#### Animal welfare mission

Study acceptability was enhanced by the participants’ perception that the animals were being well cared for. They appreciated the opportunity to assist with the animal welfare mission through care activities. One participant reflected:

I liked the way that things turned out for a lot of these animals that were inside the wild park… it showed that people… do care about these animals that are injured and… at least they've got a place to stay.

The notion of animal welfare as a *relational* facilitator reflects the connection between humans and other species and that wildlife are viewed as living beings with their own needs. Participants valued the animals and expressed concern for their well-being. Witnessing and sometimes assisting with animal care seemed to enhance participants’ appreciation of the activities. Thus, animal welfare was not only an important ethical principle but seemed to enhance acceptability of the intervention.

#### Research team

The study team had interdisciplinary expertise in human-animal interaction, health care, Veterans’ mental health, forest park management, and statistics, which provided integrated knowledge for the study plan. From an interventional perspective, the Soldier On Veteran research assistant facilitated trust and connections within the group and was instrumental in recruitment and retention. The PI, as a nurse, intentionally created a caring presence for study participants. This did not constitute providing therapy but rather a welcoming and supportive environment. Appreciation for this approach was reflected in feedback from participants that they felt “respected” and that the program was “non-judgmental.” For example, one participant who was having some psychosocial struggles, seemed unsure as to her being welcome to attend a future event. The PI told her that we would love to have her attend and hoped that she could. The following week, at the end of the activity, she gave the PI a hug and said, “I had a relapse last week and want to thank you for being part of my recovery.”

#### Responsiveness to need

Relational feasibility included being responsive to participants, community partners, study occurrences, and the evolving larger study context. While the core study design remained stable, we did make numerous minor IRB protocol modifications to address the needs of participants and partners as well as emerging contextual changes.

For example, a participant asked if they would receive a certificate after the study conclusion, so we created framed certificates of completion for participants who attended at least six of the eight activities. Participants expressed that the certificates were meaningful to them. Another modification came through a request from one of our community partners who asked to post a video of a participant activity on their social media page. Given that our partners were largely non-profit organizations with a need to demonstrate their outcomes to the public it seemed reasonable for them to highlight their work. We submitted an IRB amendment that allowed them to post photos or videos provided that the participant had given permission, and the community partner signed a form indicating that they would not identify the individual or indicate that the photo was taken during a research study.

Modifications were also made to address safety issues. For example, a mild hypoglycemic reaction experienced by a participant with Type II diabetes prompted the addition of hard candy to the first aid kit.

### Sustainability (will participants choose to integrate new patterns in their lives post-study?)

**Figure 2:**
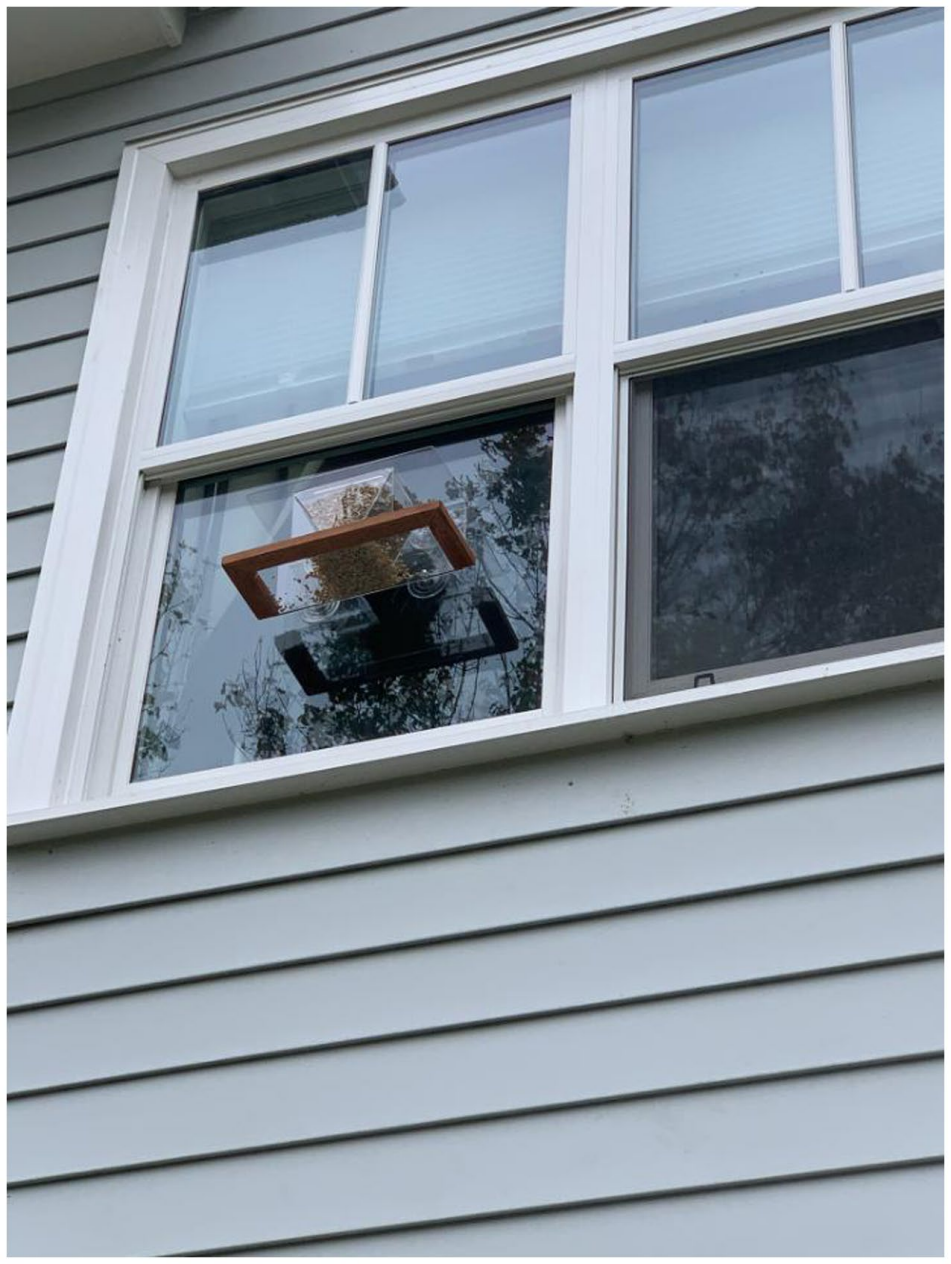
Home bird feeder.

We viewed sustainability as an ethical research principle by which participants would be provided with means to continue enjoying nature and wildlife following study conclusion. We explored three approaches to sustainability: home bird feeders, information about volunteer activities, and journalling about wildlife. The primary sustainability feature was providing participants with home bird feeders, birdseed, a bird identification book, and window decals to prevent accidental bird window strikes. The final immersion experience was focused on birds and included education on bird watching in the local region. The bird feeder provision required some navigation of residence hall rules and safety in a region with Black bears. We developed a collaborative plan with our community partner through dialogue with participants and administrators. The final plan allowed for bird feeders that could be affixed to participants’ windows with lower floor residents advised to put out birdseed only during winter months when bears were hibernating (Figure 2). Participants were provided with shelled sunflower seed to minimize ground debris. At follow up interviews, 1 month after the final activity, 7 participants had put up their bird feeders. There was an initial challenge when the feeders were beset by a flock of pigeons and one of them perched on a feeder and broke it, necessitating replacement. Eventually other bird species began visiting regularly and participants reported delight watching them. Several individuals took photos of the birds that they shared with the research assistant to send to the PI.

Additional sustainability measures included providing participants with information regarding opportunities for volunteering with local wildlife rehab facilities or participating in a citizen scientist bird count. However, at the 1 month follow up, no participants had engaged in any of these activities, despite that several had indicated a desire to do so. Several participants did indicate an expanded awareness of wildlife around them and increased knowledge about the wild animals that they saw near their homes. A post study wildlife journal activity was trialed with individuals from Cohort 1 during the Covid shutdown period. The three individuals remaining at Soldier On enrolled in this and submitted entries describing wildlife that they saw in their day-to-day lives. We also offered this option to participants in a subsequent Cohort and although they indicated interest, they did not actually submit any journal entries. Possibly the Covid shutdown period was more amenable to reflective journalling.

## Discussion

The findings suggest that recruitment and retention of Veterans with PTSD in a wildlife immersion AAI program is feasible and that the activities can be administered in a manner that is safe and acceptable with appropriate supports. This includes having nonprofessionals engage in hands-on wildlife care under supervision. We were able to recruit a diverse sample that included persons from underrepresented backgrounds as well as individuals with physical challenges. The length of the program may have been difficult for some participants to complete and shorter programs may be better suited for individuals who are navigating challenging life circumstances.

Cohorts ranged from 2 to 8 participants with varying attendance. In our experience, having 4–6 participants was most manageable. Having a larger group was a bit hectic and presented challenges finding sufficient animal care experiences at the rehab center. Conversely, a group size of 2 limited social interaction and left only one participant if the other individual was absent. The social dimension of group activities seemed to support recruitment and retention as well as contributing to building relationships among study participants that continued post study. However, it was also important to have options for individual activities for persons desiring more solitude, such as those with social anxiety. It may be helpful to examine group concordance when planning activities. Research on restorative environments suggests that social accompaniment may offer benefits such as safety, and shared discovery. However, preference for companionship varies by setting and is lower when people are experiencing attentional fatigue (45–47).

**Figure 3:**
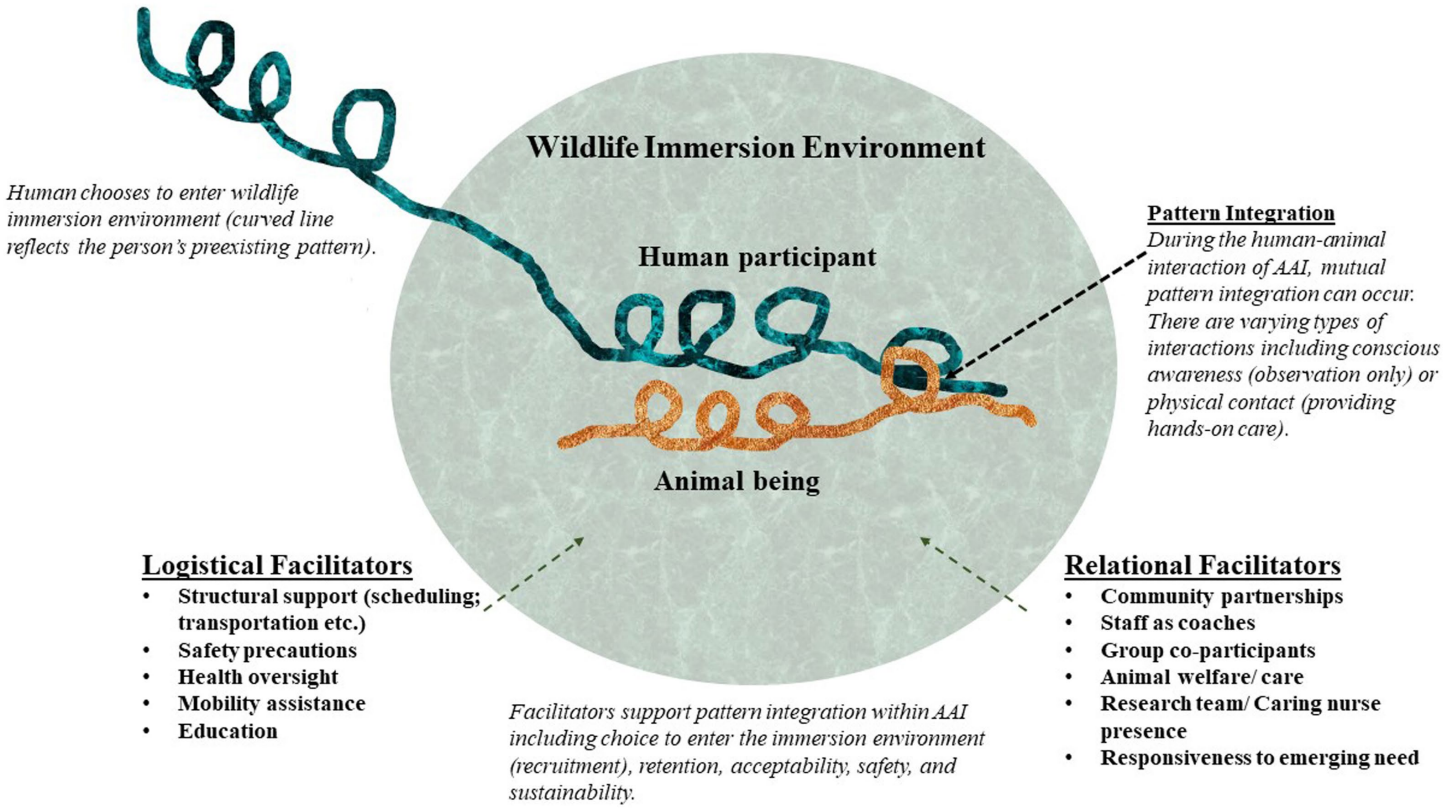
Pattern integration feasibility facilitators in AAI.

Our findings suggest that overlapping logistical and relational facilitators may enhance feasibility of a nature/wildlife immersion program. Figure 3 illustrates the facilitators supporting human-animal interactions with wildlife. The overlapping human-animal patterns in this diagram signify the occurrence of an interaction. Human-wildlife interactions include spatial, temporal, and mutually conscious dimensions (31). There are varying forms of human-wildlife interaction including physical (such as providing hands-on care) or conscious awareness (such as observation). Support needs vary with different types of human-animal interaction.

The ethical dimension of animal care was important not only for wildlife welfare but also contributed to feasibility. Animal care included both treatment and release of injured wildlife as well as sanctuary care for those that were not able to provide for themselves in the wild. Participant responses suggested that the experiences were congruent with their values about animals, and they felt that they were part of doing something good. This is consistent with studies showing that the public is increasingly concerned with captive animal welfare and many people have conflicted views towards visiting zoos (48). Animal sanctuaries, such as the one in this study, may offer an alternative as the animals are in captivity only because they require lifelong care (23, 49). Moreover, the findings related to animal welfare support the need to be attentive to the larger context of human-animal interactions and mutual wellbeing rather than a limited focus on human health benefits.

As participants engaged in these interactions, they began to integrate their own patterns with those of other species, study settings, and the natural world. Sometimes this involved transcending old patterns. For example, some participants described that having the wildlife activities each week “got us out of the house.” Immersion in the study settings helped participants to enter the patterns of natural systems in the outdoor world through activities such as walking in the woods, and observing and caring for wild creatures. Participants described gaining insight into natural patterns such as, “learning about the forest environment in New England” and “how ecosystems worked.” Patterns of wildlife care were designed to be in harmony with each animal’s nature as a wild creature. For example, one participant described, “Observing wildlife such as raccoons… The habitats were designed and constructed to resemble the environments where the animals lived.” Kaplan describes the natural environment as having a “special resonance” (50), (p. 174) with human inclinations and that the patterns of the natural world fulfill a variety of human purposes including observing other animals. Research with individuals participating in a wilderness immersion program suggested that participants experienced feelings of being ‘“at one with’” (51), (p. 182) the environment rather than feelings of control over the environment (51).

The natural environment is a dynamic system. An intervention based on immersion into a dynamic system is not a discrete fixed activity in a laboratory setting but rather an interaction within a changing environment. Some of the changes in the environment reflect rhythmical patterns such as seasonal changes. But other changes are neither regular, nor predictable (such as acorns falling or the Covid pandemic). Thus, a science of natural immersion and human-animal interaction must be able to encompass this dynamic emergence. It was interesting that when we developed an activity survey question to assess stresses in the environment, we were worried about big things like gunfire, not acorns and children. Unexpected occurrences could also make the experience extra special, such as when a large tortoise in the wildlife hospital delighted participants by unexpectedly approaching them. Immersion experiences are one possible approach to AAI in natural settings as they allow for both consistency and flexibility.

There was some variation in participant comfort level regarding engaging with particular animals. This is consistent with literature showing that humans have varied attitudes toward different species (52, 53). Emotions are an important dimension of human responses to wildlife and feelings such as fear likely have an evolutionary basis (54). Therefore, it was important to inform participants what different experiences might entail so they could choose whether or not to participate depending upon their comfort level. There was also a suggestion of developing comfort levels over time such as a participant who initially was afraid to hold the snake but, after encouragement, was able to hold it and had a meaningful experience. This is consistent with research showing that exposure to different species within a safe context can increase positive valuation of that species (23).

Intervention development can be an important aspect of pilot study work because interventions with human persons are complex and dynamic, influenced by unexpected events, and have varying meanings for participants. Hoddinott (55) notes that such studies sometimes need “further tinkering” (p. 3). Being responsive to the needs of participants and community partners reflects our theoretical framework of transcendent pluralism in which choices for the human good, or dignity, are viewed as part of a mutually evolving process. This approach is consistent with community-academic partnerships in which the expertise and needs of all partners are considered (53).

The sustainability bird feeding feature allowed participants to engage with wildlife after the study concluded, which was positively received. However, participants did not choose to engage in other post study activities, such as volunteering at rehab facilities, despite expressing strong interest and being provided with information. This suggests that providing a concrete structure for activities, as was done during the study, might be needed for some individuals to engage in wildlife pursuits. This is consistent with previous research by the PI which found that when individuals have a desire for transformative action, the desire may remain latent until a supportive structure for the activity is found (24). In post study interviews, participants described application of knowledge to interactions with wildlife in their daily lives suggesting that education is a helpful feature for sustainability.

### Limitations and strengths

Recruitment was substantially affected by the Covid pandemic. Target enrollment had been 5 cohorts with 50 total participants. However, after a 1.5-year hold, we resumed the study, and by applying safety precautions, were able to provide the intervention safely with no Covid transmission. Although recruitment from one organization greatly enhanced logistics and participant support, there were some limitations in having a more transient study population that affected retention. Sample size and recruitment from one organization also limits generalizability of the findings.

Nature is variable and sometimes unpredictable. The outdoor activities placed seasonal restrictions in a New England setting. However, future studies could explore winter wildlife activities such as finding animal tracks in snow. Additionally, the activities were based upon animal availability and needs, which meant that experiences varied. This presented some limitations in terms of ability to strictly control the intervention. However, this was also a strength in terms of a real-world evaluation. Having the PI immersed in the field with participants allowed us to be responsive to needs and capture extensive feasibility data. An additional strength was that all the interventions were conducted in settings open to the public and thus are amenable to testing with different populations and future translation. Further research is needed to extend these findings and to test results with larger sample sizes and different populations.

## Conclusion

Our study findings suggest that animal-assisted activities with wildlife, including wildlife care, are feasible for Veterans with PTSD/PTSD symptoms and concomitant physical and psychiatric conditions. Immersion in the natural world and interaction with its many species offers potential benefits to the human person. However, the natural world is a dynamic system. Research with AAI must be able to encompass dynamic emergence and complexity. An AAI involving wildlife is not an isolated prescriptive activity but influenced by multidimensional phenomena. Logistical as well as relational facilitators are important for the safe and fulfilling implementation of AAI in the natural setting. Animal welfare is an important consideration and guidance by persons knowledgeable about wildlife is critical so that human benefits do not come at the expense of other species. It is also important to consider sustainable features of AAI in order to provide individuals with ways to integrate the natural world within their daily lives. As humans learn to interact with wildlife ethically and safely, we can build a sustainable future toward the advancement of both human and wildlife wellbeing.

## Data availability statement

The datasets presented in this article are not readily available because the data are not publicly accessible. Requests to access the datasets should be directed to DonnaJ.Perry@umassmed.edu.

## Ethics statement

The studies involving humans were approved by UMass Chan Medical School Institutional Review Board. The studies were conducted in accordance with the local legislation and institutional requirements. The participants provided their written informed consent to participate in this study.

## Author contributions

DP: Conceptualization, Data curation, Formal analysis, Funding acquisition, Investigation, Methodology, Project administration, Resources, Supervision, Validation, Visualization, Writing – original draft, Writing – review & editing. SC: Conceptualization, Data curation, Formal analysis, Funding acquisition, Investigation, Methodology, Writing – original draft, Writing – review & editing. JM: Writing – review & editing, Project administration. JA: Writing – review & editing, Data curation. DS: Conceptualization, Funding acquisition, Investigation, Methodology, Project administration, Supervision, Visualization, Writing – original draft, Writing – review & editing.
