# Erratum: The feasibility of wildlife immersion experiences for Veterans with PTSD

**DOI:** 10.3389/fvets.2024.1463829

**Published:** 2024-08-09

**Authors:** Frontiers Production Office

**Affiliations:** Frontiers Media SA, Lausanne, Switzerland

**Keywords:** feasibility, human-animal interaction, human-wildlife interaction, animal-assisted intervention, Veterans, PTSD, transcendent pluralism, wildlife immersion

Due to a production error, the following sentences were omitted from the Abstract:

The theory of transcendent pluralism, which is grounded in mutual human and ecological dignity, guided the study. We viewed feasibility from the perspective of pattern integration with the natural world.

A correction has been made to the **Abstract**, “*Methods*” section:

**Methods:** This mixed methods feasibility trial involved a modified crossover study in which Veterans with PTSD/PTSD symptoms were provided a series of 8 nature and wildlife immersion experiences to evaluate feasibility and preliminary efficacy. The sample included 19 Veterans with PTSD/PTSD symptoms who were followed for a mean of 15.1 weeks. The intervention was comprised of a baseline forest walk, assisting with wildlife rehabilitation, observation in a wildlife sanctuary, and bird watching. Post study bird feeders were provided for sustainability. The theory of transcendent pluralism, which is grounded in mutual human and ecological dignity, guided the study. We viewed feasibility from the perspective of pattern integration with the natural world.

The publisher apologizes for this mistake. The original article has been updated.

